# The golden number seen in a mechanical oscillator

**DOI:** 10.1038/s41598-022-13485-7

**Published:** 2022-06-09

**Authors:** Jonatan Pena Ramirez, Erick Espinoza, Ricardo Cuesta

**Affiliations:** Center for Scientific Research and Higher Education at Ensenada (CICESE), 22860 Ensenada, BC Mexico

**Keywords:** Mechanical engineering, Applied physics, Statistical physics, thermodynamics and nonlinear dynamics

## Abstract

A seemingly ubiquitous irrational number often appearing in nature and in man-made things like structures, paintings, and physical systems, is the golden number. Here, we show that this astonishing number appears in the periodic solutions of an underactuated mass-spring oscillator driven by a nonlinear self-excitation. Specifically, by using the two-time scale perturbation method, it is analytically demonstrated that the golden number appears in the ratio of amplitudes, as well as in the oscillation frequency of the periodic solution, which is referred to as golden solution and, by applying the Poincaré method, it is demonstrated that this solution is asymptotically stable. Additionally, the analytic results are illustrated by means of numerical simulations and also, an experimental study is conducted.

## Introduction

Around 300 BC the Greek mathematician Euclid, introduced to the world the *division in extreme and mean ratio* in which, a given line is divided in two segments such that the ratio of the whole line to the larger segment is equal to the ratio of the larger segment to the shorter segment. Later, in the nineteenth century, this ratio was renamed as the golden section or golden ratio and, since then, its study has attracted the attention of mathematicians, historians, architects, among others^1^.

The numerical value of the golden ratio, which is strongly related to the Fibonacci sequence, see e.g. Ref.^2^, can be obtained as follows. Consider a line of length *x* and divide the line such that the largest segment equals 1. Then, the segments are said to satisfy the extreme and mean ratio mentioned above if the following is satisfied

1 $$\begin{aligned} x=\frac{1}{x-1}. \end{aligned}$$

Rewriting this equation yields to the second order polynomial in *x*

2 $$\begin{aligned} x^{2}-x-1=0, \end{aligned}$$

which has the roots

3 $$\begin{aligned} \varphi {:}{=}x_{+}=\frac{1+\sqrt{5}}{2}=1.61803 \ldots ,\ \ \ \ x_{-}=-\varphi ^{-1}=-0.61803 \ldots \end{aligned}$$

The positive root $\varphi$ is referred to as the golden number.

Interestingly, it has been found that the golden ratio frequently appears in nature. For example, in the structure of galaxies, in the arrangement of the petals of a rose, in the apple’s seeds, in the spiral shells of mollusks, and in the ratio of masses of two quasiparticles in cobalt niobate^3–5^, just for mentioning a few. For further examples, ranging from biology to architecture and music—including some controvertible examples on ancient buildings and paintings—the interested reader is referred to^3,6–9^.

Regarding physical systems, the golden number has appeared in, for example, a simple harmonic oscillator^10^, in the solutions of a spherical pendulum^11^, and in a network of resistors^12^.

It should be noted that, besides its intriguing geometric and mathematical interpretation, there exist examples where the golden ratio is of paramount importance. For example, a recent study^13^ has shown that the golden ratio distribution observed in the veins of tree leaves allows maximizing the bending stiffness and also, with this golden distribution, the area of the leaf exposed to the sun rays is larger, which ultimately is beneficial during the photosynthesis process. Furthermore, the golden ratio also finds interesting applications, like in the design of antennas for improving the bandwidth and increasing the antenna’s gain^14,15^, and in magnetic resonance imaging, for reducing the occurrence of eddy current related artifacts^16^, among others. In this paper, it is demonstrated that the golden ratio appears in the periodic solution of an underactuated mechanical oscillator, which is composed of two interconnected masses. Only one of the masses is excited by a weakly nonlinear term in order to have self-sustained oscillations in the system. Specifically, it is shown that the system has a stable periodic solution, which has the following properties: (1) the ratio of amplitudes corresponding to the actuated and non actuated oscillators is equal to the golden ratio, (2) the oscillation frequency of the periodic solution is equal to the natural frequency of the uncoupled oscillators scaled by the golden number. It is important to stress the fact that the aforementioned properties are independent of the intrinsic parameters of the oscillators. Furthermore, the occurrence of the golden number in the periodic solution is analytically demonstrated by using a perturbation method namely, the two-timescale method and also, analytic conditions for the stability of the periodic solution are derived by using the Poincaré method of perturbation. The obtained theoretical results are numerically illustrated and also, an experimental study is conducted in order to validate the analytic results. The experiments are performed using an electro-mechanical device.

Ultimately, the obtained analytical, numerical, and experimental results presented here give clear evidence that the underactuated mechanical oscillator with weakly nonlinear self-excitation has “golden solutions”: the golden number appears in the amplitude and frequency of the stable periodic solution.

## System description and main result

**Figure 1:**
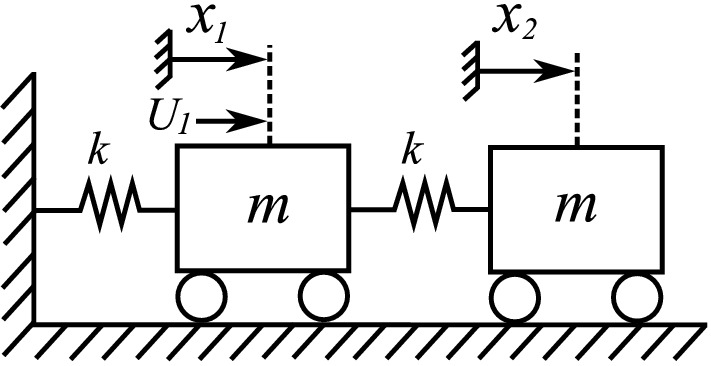
Schematic diagram of the mechanical oscillator.

Consider the mechanical system shown in Fig. 1, which consists of two identical masses of mass *m* (kg). The masses are coupled through a spring and also, one of the masses is attached to a fixed support via a spring. Both springs are identical and have stiffness coefficient *k* (N/m). Furthermore, only the first mass is actuated by the input signal $U_{1}$. Consequently, the mechanical system is indeed an underactuated system because it has more degrees of freedom than actuators, see. e.g. Refs.^17,18^.

The dynamic behavior of the system is governed by the following set of equations

4 $$\begin{aligned} \begin{aligned} \ddot{x}_{1}&=-\frac{k}{m}x_1-\frac{k}{m}(x_1-x_2)+\frac{U_{1}}{m},\\ \ddot{x}_{2}&= \frac{k}{m}(x_1-x_2), \end{aligned} \end{aligned}$$

where $x_{1},x_{2}\in {\mathbb {R}}$ describe the positions of the masses.

On the other hand, the following nonlinear term is applied to the actuated mass in order to induce a self-sustained oscillation

5 $$\begin{aligned} U_{1}=-\epsilon \lambda \left( \frac{1}{2}m{\dot{x}}_{1}^{2}+\frac{1}{2}kx_{1}^{2}-H^{*}\right) {\dot{x}}_{1}, \end{aligned}$$

where $0<\epsilon \ll 1$ is a small parameter and $\lambda ,H^{*}\in {\mathbb {R}}_{+}$ are design parameters. Note that the nonlinear term (5) dissipates energy for $(\frac{1}{2}m{\dot{x}}_1^2+\frac{1}{2}kx_{1}^2)>H^{*}$, whereas it pumps energy into the system for $(\frac{1}{2}m{\dot{x}}_1^2+\frac{1}{2}kx_{1}^2)<H^{*}$, see e.g. Ref.^19^.

### Determining the golden periodic solution in the system

In order to unveil the presence of the golden number in the amplitude and frequency of the periodic solution in system (4)–(5), we use the two-timescales method, also known as two-timing method, see e.g. Refs.^20–23^.

First, let define the dimensionless time

6 $$\begin{aligned} \tau =\omega t,\ \ \ \ \ \ \ \omega =\sqrt{\frac{k}{m}}, \end{aligned}$$

such that system (4)–(5) can be written in the form

7 $$\begin{aligned} \begin{aligned} \ddot{x}_{1}&=-x_1-(x_{1}-x_{2})-\epsilon p_{1}(p_{2}{\dot{x}}^{2}_{1}+p_{2} x_{1}^{2}-H^{*}){\dot{x}}_{1}, \\ \ddot{x}_{2}&=(x_{1}-x_{2}), \end{aligned} \end{aligned}$$

where the over-dots denote differentiation with respect to $\tau$ and

8 $$\begin{aligned} p_{1}=\frac{\lambda }{\omega m},\ \ \ \ \ \text{ and }\ \ \ \ \ p_{2}=\frac{1}{2}k. \end{aligned}$$

The next step is to look for solutions of (7) in the form

9 $$\begin{aligned} x_{1}(\tau )=-ax_{2}(\tau ), \quad \text{ with }\quad a\in {\mathbb {R}}_{+}. \end{aligned}$$

Now, the two-timescale method is used in order to obtain explicit expressions for $x_{1},x_{2}$, and *a*. In accordance with this well-known perturbation method and considering (9), the following ansatz is proposed

10 $$\begin{aligned} \begin{aligned} x_{1}(\tau )&=-ax_{01}(\tau ,T)-a\epsilon x_{11}(\tau ,T)+{\mathscr {O}}(\epsilon ^2),\\ x_{2}(\tau )&=x_{01}(\tau ,T)+\epsilon x_{11}(\tau ,T)+{\mathscr {O}}(\epsilon ^2). \end{aligned} \end{aligned}$$

where $\tau$ is the fast time and $T=\epsilon \tau$ is the slow time. Note that (10) is indeed and asymptotic approximation under the assumption that $\epsilon \ll 1$.

The first and second derivatives of (10) with respect to $\tau$ are given by

11 $$\begin{aligned} \begin{aligned} {\dot{x}}_{1}&=-ax'_{01}-a\epsilon (x'_{11}+\frac{\partial }{\partial T}x_{01})+{\mathscr {O}}(\epsilon ^{2}), \ \ {\dot{x}}_{2}=x'_{01}+\epsilon (x'_{11}+\frac{\partial }{\partial T}x_{01}) +{\mathscr {O}}(\epsilon ^{2}),\\ \ddot{x}_{1}&=-ax''_{01}-a\epsilon (x''_{11}+2\frac{\partial }{\partial T}x'_{01})+{\mathscr {O}}(\epsilon ^{2}), \ \ \ddot{x}_{2}=x''_{01}+\epsilon (x''_{11}+2\frac{\partial }{\partial T}x'_{01})+{\mathscr {O}}(\epsilon ^{2}), \end{aligned} \end{aligned}$$

where the primes indicate partial differentiation with respect to $\tau$, that is $x'=\frac{\partial x}{\partial \tau }$.

Replacing the approximated solution (10) and its derivatives (11) into the first equation of (7), and ignoring high order terms in $\epsilon$ $(\ge \epsilon ^{2})$, yields to

12 $$\begin{aligned} x''_{01}+\epsilon (x''_{11}+2\frac{\partial }{\partial T}x'_{01})=-\frac{2a+1}{a}x_{01}-\frac{2a+1}{a}\epsilon x_{11}-\epsilon p_1(p_2a^{2}{x'_{01}}^{2}+p_2a^{2}x_{01}^2-H^{*})x'_{01}. \end{aligned}$$

Before solving (12), it is convenient to re-scale the time according to

13 $$\begin{aligned} {\bar{\omega }}=\sqrt{\frac{2a+1}{a}},\ \ \ \ \ \ \ {\bar{\tau }}={\bar{\omega }}\tau . \end{aligned}$$

such that Eq. (12) is rewritten as follows

14 $$\begin{aligned} x''_{01}+\epsilon (x''_{11}+\frac{2}{{\bar{\omega }}}\frac{\partial }{\partial T}x'_{01})=-x_{01}-\epsilon x_{11} -\epsilon \frac{p_1}{{\bar{\omega }}}(p_2a^{2}{\bar{\omega }}^{2}{x'_{01}}^2+p_2a^{2}x_{01}^2-H^{*})x'_{01}, \end{aligned}$$

where now the primes indicate partial differentiation with respect to time ${\bar{\tau }}$.

By collecting terms with similar powers in $\epsilon$, it is possible to obtain the following set of equations

15 $$O(\epsilon ^0)= O(1): \ x''_{01}+x_{01}= 0,$$

16 $$O(\epsilon ^1)= O(\epsilon ): \ x''_{11}+x_{11}=-\frac{2}{{\bar{\omega }}}\frac{\partial }{\partial T} x'_{01}-\frac{p_1}{{\bar{\omega }}}(p_2a^{2}{\bar{\omega }}^2 {x'_{01}}^2+p_2a^{2}x_{01}^2-H^{*})x'_{01}.$$

The general solution of (15) is

17 $$\begin{aligned} x_{01}({\bar{\tau }},T)=A(T)\cos {({\bar{\tau }}+\theta (T))}, \end{aligned}$$

where *A*(*T*) is the amplitude of the solution and $\theta (T)$ its phase. This result can be used for solving (16). First, note that

18 $$x'_{01}=\frac{\partial }{\partial {\bar{\tau }}}x_{01}=-A(T)\sin {({\bar{\tau }}+\theta (T))}$$

19 $$\frac{\partial }{\partial T}x'_{01}=-{\dot{A}}\sin {({\bar{\tau }}+\theta )}-A{\dot{\theta }}\cos {({\bar{\tau }}+\theta (T))},$$

where the over-dots denote differentiation with respect to *T*.

Replacing (18) and (19) into (16) yields to

20 $$\begin{aligned} x''_{11}+x_{11}=h({\bar{\tau }}), \end{aligned}$$

with

21 $$\begin{aligned} h({\bar{\tau }})=\frac{2}{{\bar{\omega }}}({\dot{A}}\sin {({\bar{\tau }}+\theta )}+A{\dot{\theta }}\cos {({\bar{\tau }}+\theta ))}+P, \end{aligned}$$

where $$P=\frac{p_1}{{\bar{\omega }}}(p_2a^{2}A^2({\bar{\omega }}^2\sin ^2{({\bar{\tau }}+\theta )}+\cos ^2{({\bar{\tau }}+\theta )})-H^{*})A\sin {({\bar{\tau }}+\theta )}.$$

In order to continue with the analysis, it is worth to remember the following result from the theory of differential equations^23^:

#### Proposition 1

*Let* $h({\bar{\tau }})$ *be a* $2\pi$-*periodic function of* ${\bar{\tau }}$. *Let* $\theta$ *be any fixed real number*. *Then, the equation* $\ddot{x}+x=h$ *has a* $2\pi$-*periodic solution* *x* *if and only if*

22 $$\begin{aligned} \int _{0}^{2\pi }h({\bar{\tau }})\sin ({\bar{\tau }}+\theta )\text{ d }{\bar{\tau }}=0 \ \ \ \ \ \ \text{ and } \ \ \ \ \ \ \int _{0}^{2\pi }h({\bar{\tau }})\cos ({\bar{\tau }}+\theta )\text{ d }{\bar{\tau }}=0. \end{aligned}$$

Since function $h({\bar{\tau }})$ given in (21) is indeed $2\pi$-periodic, we can make use of Proposition 1. Then, replacing (21) in (22), and solving the integrals yields to

23 $$\begin{aligned} \int _{0}^{2\pi }h({\bar{\tau }})\sin ({\bar{\tau }}+\theta )\text{ d }{\bar{\tau }} =&3p_{1}p_{2}A^{3}a^{2}{\bar{\omega }}^{2}+p_{1}p_{2}a^{2}A^{3}-4H^{*}p_1A+8{\dot{A}}= 0, \end{aligned}$$

24 $$\begin{aligned} \int _{0}^{2\pi }h({\bar{\tau }})\cos ({\bar{\tau }}+\theta )\text{ d }{\bar{\tau }} =&\frac{2\pi A{\dot{\theta }}}{{\bar{\omega }}}=0. \end{aligned}$$

From (23) it is possible to obtain the following differential equation

25 $$\begin{aligned} {\dot{A}}=\frac{1}{8}A[4H^{*}p_1-a^{2}p_{1}p_{2}(3{\bar{\omega }}^2+1)A^2], \end{aligned}$$

which has the following equilibrium points

26 $$\begin{aligned} A^{\star }=0, \ \ \ A^{\star }_{+}=\sqrt{\frac{4H^{*}}{a^{2}p_{2}(3{\bar{\omega }}^2+1)}},\ \ \ \ A^{\star }_{-}=-\sqrt{\frac{4H^{*}}{a^{2}p_{2}(3{\bar{\omega }}^2+1)}}. \end{aligned}$$

The equilibrium point $A^{\star }$ is unstable, whereas the equilibriums $A^{\star }_{+}$ || $A^{\star }_{-}$ are stables. Since *A* represents the amplitude of the periodic solution, see Eq. (17), we will focus, without lose of generality, on the positive equilibrium $A^{\star }_{+}$. Then, by linearizing (25) around $A^{\star }_{+}$ it can be easily shown that the limit solution of (25) satisfies

27 $$\begin{aligned} \lim _{T\rightarrow \infty }A=A^{\star }_{+}=\sqrt{\frac{4H^{*}}{a^{2}p_{2}(3{\bar{\omega }}^2+1)}}. \end{aligned}$$

On the other hand, from (24) it follows that ${\dot{\theta }}$ should satisfy

28 $$\begin{aligned} {\dot{\theta }}=0, \end{aligned}$$

which implies that $\theta$ is a constant, i.e.

29 $$\begin{aligned} \theta (T)=\theta _0, \end{aligned}$$

with $\theta _0\in {\mathbb {R}}$ being an arbitrary constant.

The next step is to replace (27) and (29) in the approximated solution (17), which results in

30 $$\begin{aligned} x_{01}({\bar{\tau }},T)=A^{\star }_{+}\cos {({\bar{\tau }}+\theta _{0})}. \end{aligned}$$

Now, by substitution of (30) into the approximated solutions (10), and transforming from time ${\bar{\tau }}$ back to $\tau$ using (13), yields to

31 $$\begin{aligned} x_{1}(\tau )= & {} -aA^{\star }_{+}\cos {\left( \sqrt{\frac{(2a+1)}{a}}\tau +\theta _{0}\right) }+{\mathscr {O}}(\epsilon ), \end{aligned}$$

32 $$\begin{aligned} x_{2}(\tau )= & {} A^{\star }_{+}\cos {\left( \sqrt{\frac{(2a+1)}{a}}\tau +\theta _{0}\right) }+{\mathscr {O}}(\epsilon ), \end{aligned}$$

with $A^{\star }_{+}$ given in (27). Note that the approximated solutions (31)–(32) satisfy (9).

The remaining task at this point is to find the value of *a* in (31)–(32). For this, the second derivative of (32) is computed, which results in

33 $$\begin{aligned} \ddot{x}_{2}=-\frac{2a+1}{a}A_{+}^{\star }\cos {\left( \sqrt{\frac{(2a+1)}{a}}\tau +\theta _{0}\right) }. \end{aligned}$$

Next, substitute (31)–(33) into the second equation of (7) to obtain

34 $$\begin{aligned} -\frac{2a+1}{a}A^{\star }_{+}\cos {\left( \cdot \right) }= -aA^{\star }_{+}\cos {\left( \cdot \right) }-A^{\star }_{+}\cos {\left( \cdot \right) }, \end{aligned}$$

where the argument of the cosine function is the same than in (32). In order to obtain the value of *a*, it is convenient to rewrite (34) in the form

35 $$\begin{aligned} \left( a^{2}-a-1\right) A^{\star }_{+}\cos {\left( \cdot \right) }=0. \end{aligned}$$

Clearly, for satisfying (35) for all time $\tau$ it is required that

36 $$\begin{aligned} a^{2}-a-1=0. \end{aligned}$$

Note that this polynomial in *a* is exactly the same than the polynomial from which the golden number is obtained, see Eq. (36). Therefore, as already mentioned in (3), the values of *a* satisfying (36) are

37 $$\begin{aligned} a_{1}=\varphi =\frac{1+\sqrt{5}}{2},\ \ \ \ \ a_{2}=\frac{1-\sqrt{5}}{2}. \end{aligned}$$

However, since *a* should be a positive number, see (9), only the positive root of (36) is considered and therefore we have that $a=\varphi {:}{=}\frac{1+\sqrt{5}}{2},$ i.e. the parameter *a* appearing in the amplitude and frequency of solutions (31)-(32) is the **golden number**.

Then, by substituting $a=\varphi$, (6), and (26) into (31)–(32), it follows that the approximated solution of system (4)–(5) is

38 $$\begin{aligned} x_{1}(t)= & {} -\varphi \sqrt{\frac{4H^{*}}{\varphi ^{2}p_2(3{\bar{\omega }}^2+1)}}\cos {\left( \varphi \sqrt{\frac{k}{m}}t+\theta _{0}\right) }, \end{aligned}$$

39 $$\begin{aligned} x_{2}(t)= & {} \sqrt{\frac{4H^{*}}{\varphi ^{2}p_2(3{\bar{\omega }}^2+1)}}\cos {\left( \varphi \sqrt{\frac{k}{m}} t+\theta _{0}\right) }. \end{aligned}$$

Finally, note that the above expressions can be further simplified by replacing $p_{1}$ and $p_{2}$ as given in (8) and ${\bar{\omega }}$ given in (13). This yields

40 $$\begin{aligned} x_{1}(t)= & {} -\varphi \sqrt{\frac{2H^{*}}{(3+\frac{5}{4}\sqrt{5})k}}\cos {\left( \varphi \sqrt{\frac{k}{m}}t+\theta _{0}\right) }, \end{aligned}$$

41 $$\begin{aligned} x_{2}(t)= & {} \sqrt{\frac{2H^{*}}{(3+\frac{5}{4}\sqrt{5})k}}\cos {\left( \varphi \sqrt{\frac{k}{m}} t+\theta _{0}\right) }. \end{aligned}$$

Clearly, the golden number $\varphi$ appears in the ratio of amplitudes and likewise, the oscillation frequency of the solution is equal to the natural frequency $\omega =\sqrt{k/m}$ scaled by the golden number.

### Stability of the golden solution

In this part, the stability of the golden solutions (38)–(39) is investigated by using the Poincaré method of perturbation^24^.

As a first step, system (7) is rewritten in the weakly nonlinear form

42 $$\begin{aligned} {\dot{x}}=Ax+\epsilon F(x), \end{aligned}$$

where $$x=\left[ \begin{array}{cccc}x_{1}&{\dot{x}}_{1}&x_{2}&{\dot{x}}_{2}\end{array}\right] ^{T}$$, and

43 $$\begin{aligned}&A=\left[ \begin{array}{cccc} 0&{}1&{}0&{}0\\ -{\bar{\omega }}^{2}&{}0&{}0&{}0\\ 0&{}0&{}0&{}1\\ 0&{}0&{}-{\bar{\omega }}^{2}&{}0 \end{array}\right] ,\nonumber \\&F(x)=\left[ \begin{array}{c} 0\\ \gamma (-2x_{1}+x_{2}+{\bar{\omega }}^{2}x_{1})-p_{1}h_{1}\dot{x_{1}}\\ 0\\ \gamma (x_1-x_2+{\bar{\omega }}^{2}x_{2}) \end{array}\right] , \end{aligned}$$

with

44 $$\begin{aligned} \gamma =\frac{1}{\epsilon },\ \ \ \ h_{1}=p_{2}({\dot{x}}_{1}^{2}+x_{1}^{2})-H^{*}. \end{aligned}$$

It is important to note that in (42) we have added and subtracted the term ${\bar{\omega }}^2 x_{1}$ in the first equation of (7) and also the term ${\bar{\omega }}^{2}x_{2}$ to the second equation. This may seem artificial, but in this way, we guarantee that for $\mu =0$, the systems has the desired oscillation frequency. Furthermore, the obtained results will only be valid for that specific frequency.

Next, system (42) is diagonalized using the transformation

45 $$\begin{aligned} x=Vz,\ \ \ \ \ \ \ \ V= \left[ \begin{array}{cccc} -\frac{i}{a} &{} \frac{i}{a}&{} 0&{} 0\\ 1&{} 1&{} 0&{} 0\\ 0&{} 0&{} -\frac{i}{a}&{} \frac{i}{a}\\ 0&{} 0&{} 1&{} 1\end{array}\right] , \end{aligned}$$

where *V* is the matrix of eigenvectors associated to matrix *A*, see (43), and $i=\sqrt{-1}$. Then, in the new coordinates $z\in {\mathbb {R}}^{4}$, system (42) takes the form

46 $$\begin{aligned} {\dot{z}}=Dz+\epsilon V^{-1}F(Vz),\ \ \ \ D=\left[ \begin{array}{cccc} i{\bar{\omega }} &{} 0&{} 0&{} 0\\ 0&{} -i{\bar{\omega }}&{} 0&{} 0\\ 0&{} 0&{} i{\bar{\omega }}&{}0\\ 0&{} 0&{} 0&{} -i{\bar{\omega }}\end{array}\right] . \end{aligned}$$

Note that for $\epsilon =0$, the asymptotic solutions of (46) are

47 $$\begin{aligned} z_{1}(\tau )=\alpha _{1}e^{i{\bar{\omega }}\tau }, \ \ z_{2}(\tau )=\alpha _{2}e^{-i{\bar{\omega }}\tau },\ \ z_{3}(\tau )=\alpha _{3}e^{i{\bar{\omega }}\tau }, \ \ z_{4}(\tau )=\alpha _{4}e^{-i{\bar{\omega }}\tau }, \end{aligned}$$

where $\alpha _{i}$ are arbitrary constants.

However, for $\epsilon \ne 0$, the interest is in finding a unique set of $\alpha _{i}$’s such that the obtained solution exists and is asymptotically stable. This can be done by using Theorem 1 presented in Ref.^24^. That theorem establishes that the unique set of $\alpha _{i}$’s can be obtained by solving the following periodicity conditions

48 $$\begin{aligned}&Q_{1}=-\alpha _{4}P_{1}-\alpha _{1}P_{4}=0, \end{aligned}$$

49 $$\begin{aligned}&Q_{2}=-\alpha _{4}P_{2}+\alpha _{2}P_{4}=0, \end{aligned}$$

50 $$\begin{aligned}&Q_{3}=-\alpha _{4}P_{3}-\alpha _{3}P_{4}=0, \end{aligned}$$

where the functions $P_{j}$, $j=1,\ldots ,4$, are computed from

51 $$\begin{aligned} P_{j}=\int _{0}^{2\pi /{\bar{\omega }}}f_{j}\left( \alpha _{1}e^{i{\bar{\omega }}\tau },\alpha _{2}e^{-i{\bar{\omega }}\tau },\alpha _{3}e^{i{\bar{\omega }}\tau },\alpha _{4}e^{-i{\bar{\omega }}\tau }\right) e^{-in_{j}{\bar{\omega }}\tau }\text{ d }\tau ,\ \ \ \end{aligned}$$

where $n_{1}=n_{3}=1$, $n_{2}=n_{4}=-1$, and $f_{j}(\cdot )$ is the *jth* entry of vector $V^{-1}F(Vz)$, which is described in (46). Solving (51) yields

52 $$\begin{aligned} P_{1}= & {} \frac{-\pi }{a^{3}}\left[ \gamma a\left( \alpha _{1}(a^{2}-2)+\alpha _{3}\right) i+p_{1} a^{2}\alpha _{1}\left( 3\alpha _{1}\alpha _{2}p_{2}-H^{*}\right) +\alpha _{1}^{2}\alpha _{2}p_{1}p_{2}\right] ,\ \ \ \ \end{aligned}$$

53 $$\begin{aligned} P_{2}= & {} \frac{\pi }{a^{3}}\left[ \gamma a\left( a^{2}\alpha _{2}+2\alpha _{2}-\alpha _{4}\right) i+p_{1} a^{2}\alpha _{2}\left( 3\alpha _{1}\alpha _{2}p_{2}-H^{*}\right) -\alpha _{1}\alpha _{2}^{2}p_{1}p_{2}\right] ,\ \ \ \end{aligned}$$

54 $$\begin{aligned} P_{3}= & {} \frac{\pi \gamma }{a^{2}}\left[ \alpha _{1}+\alpha _{3}\left( a^{2}-1\right) \right] i, \end{aligned}$$

55 $$\begin{aligned} P_{4}= & {} \frac{\pi \gamma }{a^{2}}\left[ \alpha _{2}+\alpha _{4}\left( a^{2}-1\right) \right] i, \end{aligned}$$

where the lowercase $p_{i}$’s are as given in (8).

After long but straightforward computations it can be shown that the values of $\alpha _{i}$’s satisfying (48)–(50) are

56 $$\begin{aligned} \alpha _{1}=\alpha _{2}=-\frac{a{\bar{w}}}{2}\sqrt{\frac{4H^{*}}{ap_{2}(3{\bar{\omega }}^{2}+1)}},\ \ \ \alpha _{3}=\alpha _{4}=-\frac{{\bar{w}}}{2}\sqrt{\frac{4H^{*}}{ap_{2}(3{\bar{\omega }}^{2}+1)}}, \end{aligned}$$

where *a* is the solution of (36).

Note that in the original coordinates *x*, the solutions (47), (56) exactly coincide with those already obtained in (38)–(39) with $\theta _{0}=-\pi /2$. This can be easily shown by replacing (47), (56) into (45) with $a=\varphi$ and using (13).

As a next step, the stability of solutions (47), (56) is investigated using a result based on the Poicaré method of perturbation. Specifically, the stability of the solutions is determined using again Theorem 1 in^24^. Following that theorem, yields to the characteristic polynomial

57 $$\begin{aligned} p(\lambda )=\text{ det }\left( \left. \left[ \begin{array}{ccc} \frac{\partial Q_{1}}{\partial \alpha _{1}}&{}\frac{\partial Q_{1}}{\partial \alpha _{2}}&{}\frac{\partial Q_{1}}{\partial \alpha _{3}}\\ \frac{\partial Q_{2}}{\partial \alpha _{1}}&{}\frac{\partial Q_{2}}{\partial \alpha _{2}}&{}\frac{\partial Q_{2}}{\partial \alpha _{3}}\\ \frac{\partial Q_{3}}{\partial \alpha _{1}}&{}\frac{\partial Q_{3}}{\partial \alpha _{2}}&{}\frac{\partial Q_{3}}{\partial \alpha _{3}}\end{array}\right] \right| _{\alpha _{i}=\alpha ^{*}_{i}}+\alpha _{4}\lambda \left[ \begin{array}{ccc}1&{}0&{}0\\ 0&{}1&{}0\\ 0&{}0&{}1\end{array}\right] \right) =0, \end{aligned}$$

where $\alpha _{i}^{*}$, $i=1,2,3$, are the values of the $\alpha _{i}$’s given in (56). Then, according to Theorem 1 in Ref.^24^, the periodic solutions (47)–(56) are asymptotically stable if and only if all the roots of the characteristic polynomial (57) have negative real parts.

In order to determine this, let replace (48)–(50), with $P_{j}$, $j=1,\ldots ,4$, as given (52)–(55), into (57). This yields the polynomial

58 $$\begin{aligned} p(\lambda )=\lambda ^{3}+a_{2}\lambda +a_{1}\lambda +a_{0}=0, \end{aligned}$$

where

59 $$\begin{aligned} a_{2}= & {} \frac{2\pi H^{*}p_{1}}{a}, \end{aligned}$$

60 $$\begin{aligned} a_{1}= & {} \frac{\gamma ^2\pi ^2}{a^4}(3a^2 - 3a + 2), \end{aligned}$$

61 $$\begin{aligned} a_{0}= & {} \frac{\gamma ^2\pi ^3}{a^6}(\gamma (1 - a - 3a{^2} + 2a{^3})i + 2aH^{*}p_{1}(1+a^2)). \end{aligned}$$

At this point it is worth mentioning that *a* can only take two possible values, see Eq. (37). For any of these two values, the term $(3a^2 - 3a + 2)$ in (60) satisfies $(3a^2 - 3a + 2)=5$, and likewise, the imaginary term in (61) vanishes. Consequently, the coefficients (60)–(61) are reduced to

62 $$\begin{aligned} a_{1}= & {} \frac{5\gamma ^2\pi ^2}{a^4}, \end{aligned}$$

63 $$\begin{aligned} a_{0}= & {} \frac{\gamma ^2\pi ^3}{a^5}2H^{*}p_{1}(1+a^2). \end{aligned}$$

By straightforward computations, it is possible to show that all the roots of the characteristic polynomial (58) with coefficient $a_{2}$ given in (59), and coefficients $a_{1}$ and $a_{0}$ as given in (62)–(63) respectively, will have negative real parts if and only if the following condition is satisfied

64 $$\begin{aligned} \frac{2H^{*}p_1\gamma ^2\pi ^{3}}{a^{5}}(4a^{2} - 1))>0. \end{aligned}$$

Hence, if $a=a_{2}=\frac{1-\sqrt{5}}{2}$, see Eq. (37), then the stability condition (64) is not satisfied and therefore the corresponding solution is *unstable*. On the other hand, if $a=a_{1}=\frac{1+\sqrt{5}}{2}{:}{=}\varphi$, see Eq. (37), the condition (64) is satisfied and then, according to Theorem 1 in Ref.^24^, the solution (47), (56) is *asymptotically stable*. Furthermore, since the transformation (45) is nonsingular, the stability conditions of the periodic solutions (47), (56) also hold for the solutions in the original coordinates, i.e. for solutions (40)–(41).

Summarizing, the above results provide analytic evidence that the golden number appears in the amplitude and frequency of the periodic solution of system (4) and that this solution is asymptotically stable. The analysis has been conducted using two perturbation methods namely, the two-timing and the Poincaré methods. The obtained results are formalized in the following theorem.

#### Theorem 1

*Consider the dynamical system described by* (4). *Assume that the actuated part of the system is driven by the nonlinear term* (5) *with* $0<\epsilon \ll 1$. *Then, the approximated periodic solutions* (${\mathscr {O}}(\epsilon )$) *of system* (4)–(5) *have the form*

65 $$\begin{aligned} x_{1}(t)&=-\varphi A_{+}^{*}\cos {\left( \varphi \omega t+\theta _{0}\right) }+{\mathscr {O}}(\epsilon ),\\ x_{2}(t)&=A_{+}^{*}\cos {\left( \varphi \omega t+\theta _{0}\right) }+{\mathscr {O}}(\epsilon ), \end{aligned}$$

*where* $\varphi \in {\mathbb {R}}_{+}$ *is the* *golden number*, $\omega =\sqrt{\frac{k}{m}}\in {\mathbb {R}}_{+}$ *is the natural frequency*, $\theta _{0}\in {\mathbb {R}}_{+}$ *is an arbitrary phase shift, and*

66 $$\begin{aligned} A_{+}^{\star }=\sqrt{\frac{2H^{*}}{(3+\frac{5}{4}\sqrt{5})k}}\ \end{aligned}$$

*where* $H^{*}$ *is a design parameter of the nonlinear excitation* (5) *and* $k\in {\mathbb {R}}_{+}$ *is the stiffness*. *Due to the fact that the golden number appears in the ratio of amplitudes and also in the oscillation frequency, solutions* (65) *are referred to as* *golden solutions*. *Furthermore, these solutions are asymptotically stable*.

#### Remark 1

The golden number appears in the ratio of amplitudes of the approximated periodic solutions only if the oscillators are **identical**. Thus, the golden number can be used a measure of how identical or dissimilar the oscillators are.

## The golden solutions and the energy in the system

This section highlights the importance of the golden solutions (65) from an energy viewpoint. For this, it is convenient to use the theory of passive systems and consider the following definition, which has been derived following^25^.

### Definition 1

Consider system (4) with input $U_{1}$ and output $y={\dot{x}}_{1}$, i.e. the output of the system is the velocity of the first oscillator. Then, the system is said to be **lossless**, i.e. with no energy dissipation, if there exists a continuously differentiable positive semidefinite function *V*(*x*) (called the storage function) such that

67 $$\begin{aligned} {\dot{V}}=U_{1}y. \end{aligned}$$

For the present case, let consider the following storage function

68 $$\begin{aligned} V=\frac{1}{2}m{\dot{x}}_{1}^{2}+\frac{1}{2}kx_{1}^{2}+\frac{1}{2}m{\dot{x}}_{2}^{2}+\frac{1}{2}k(3a+2)x_{2}^{2}. \end{aligned}$$

The time derivative of this storage function is

69 $$\begin{aligned} {\dot{V}}=m{\dot{x}}_{1}\ddot{x}_{1}+kx_{1}{\dot{x}}_{1}+m{\dot{x}}_{2}\ddot{x}_{2}+k(3a+2)x_{2}{\dot{x}}_{2}. \end{aligned}$$

By replacing (4) into (69) and using (9), we obtain

70 $$\begin{aligned} {\dot{V}}=(-a^{2}+a+1)kx_{2}{\dot{x}}_{2}+U_{1}y. \end{aligned}$$

Therefore, if $a=\varphi$ or $a=-\frac{1}{\varphi }$, see (3), then (70) reduces to (67) and consequently, it follows from Definition 1 that system (4) is lossless.

### Remark 2

Note that there are two possible values of *a* for which $(-a^2+a+1)=0$ in (70). However, as demonstrated in Theorem 1, the only stable periodic solution satisfying (9) is obtained for $a=\varphi$. Thus, the golden solution (65) is important from an energy viewpoint because for that particular solution there is no dissipation in the system.

## Numerical study

In this section, the analytic results summarized in Theorem 1 are illustrated by means of numerical simulations. Hence, system (4)–(5) is numerically integrated using the Runge Kutta method implemented in Matlab with a step size of $1\times 10^{-3}$. The parameter values are summarized in Table 1 and the initial condition $x_{1}(0)$ is arbitrarily chosen as $x_{1}(0)=5\times 10^{-3}$ (m), and the remaining initial conditions are set to zero.

**Table 1:** Parameter values of system (4)–(5). These parameter values have been obtained partly from the experimental setup ECP MODEL 210 discussed in the next section.

Parameter	Value	Units
*k*	463.74	N/m
*m*	2.77	kg
$\epsilon$	0.01	–
$\lambda$	600	s/kg m$^2$
$H^{*}$	0.06	Nm

**Figure 2:**
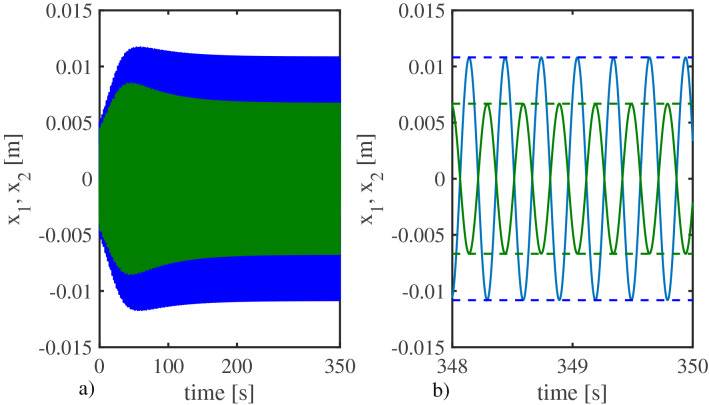
Numerical results. (**a**) Complete time series for $x_{1}$ (blue), and $x_{2}$ (green). (**b**) Zoom-in into the last 2 s of the simulation (steady behavior). The horizontal blue and green dashed lines indicate the theoretical values of the amplitudes predicted by Theorem 1, see (65)–(66).

Figure 2a shows the complete time series corresponding to the positions of the oscillators, denoted by $x_{1}$ (in blue) and $x_{2}$ (in green), whereas Figure 2b shows a zoom in the last two seconds of the simulation (steady behavior).

**Figure 3:**
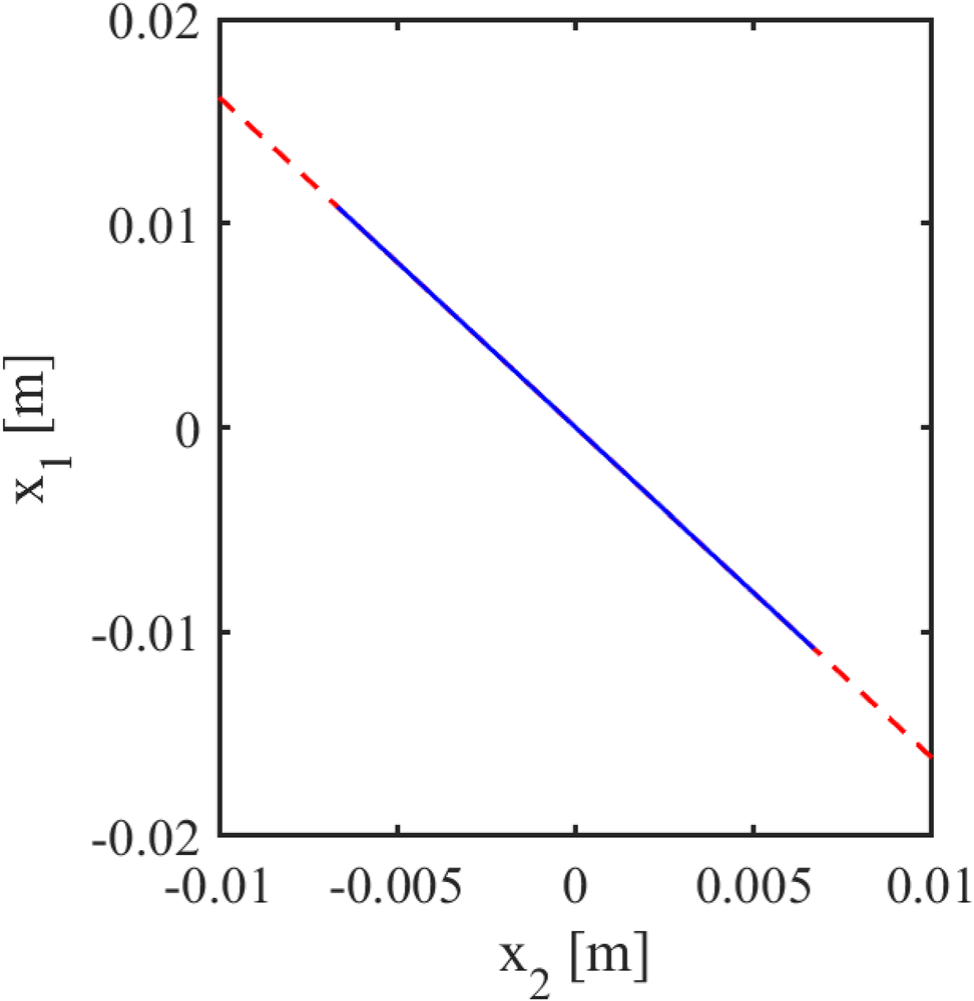
Numerical results. Projection of the solutions of system (4)–(5) onto the ($x_{2},x_{1}$)-plane, indicated by the blue line. The dotted red line has slope $-\varphi$ and is just for reference. Thus, it is clear from this figure that the golden number $\varphi$ appears in the ratio of amplitudes in the solutions of the system.

For the parameter values considered in this simulation, the expected values for the amplitudes, obtained using (65), (66), are $x_{1amp}=\varphi A_{+}^{*}=0.0108$ and $x_{2amp}=A_{+}^{*}=0.0067$. These values are indicated by the horizontal blue and green dashed lines respectively, in Fig. 2b. On the other hand, the amplitudes obtained from the simulation (solid blue and green signals) are $x_{1amp}=\varphi A_{+}^{*}=0.01081$ and $x_{2amp}=A_{+}^{*}=0.00668$. Clearly, there is a good agreement between the theoretical and numerical results. Consequently, these numerical results confirm that the ratio of amplitudes coincides indeed with the golden number. This can be further verified from Fig. 3, which shows the projections of the solutions of system (4)–(5) onto the ($x_{2},x_{1}$)-plane, see the blue line. The dotted red line is just a reference line with slope $-\varphi$.

**Figure 4:**
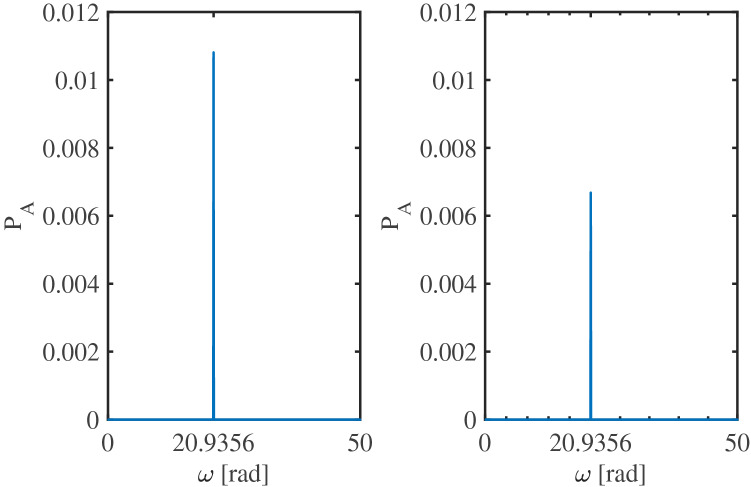
Single-sided amplitude spectrum for the solutions $x_{1}$ (left) and $x_{2}$ (right) of system (4)–(5). The vertical axis indicates the peak amplitude $P_{A}$ of the corresponding signals. From this figure it is clear to see that the frequency of the solutions is $\varphi \omega =\varphi \sqrt{\frac{k}{m}}=20.9356$ (rad), which corresponds to the natural frequency scaled by the golden number.

Finally, Fig. 4 shows the single-sided amplitude spectrum for solutions $x_{1}$ and $x_{2}$. Clearly, the oscillation frequency of the solutions is at $\varphi \omega =\varphi \sqrt{\frac{k}{m}}=20.9356$ (rad). Note that the oscillation frequency is indeed equal to the natural frequency scaled by the golden number, just as indicated by Theorem 1.

In summary, the numerical results presented here give clear evidence that the golden number appears in the amplitude and in the frequency of the periodic solutions of system (4)–(5).

## Experiments

This section presents and experimental study in order to validate the theoretical and numerical results obtained in previous sections.

**Figure 5:**
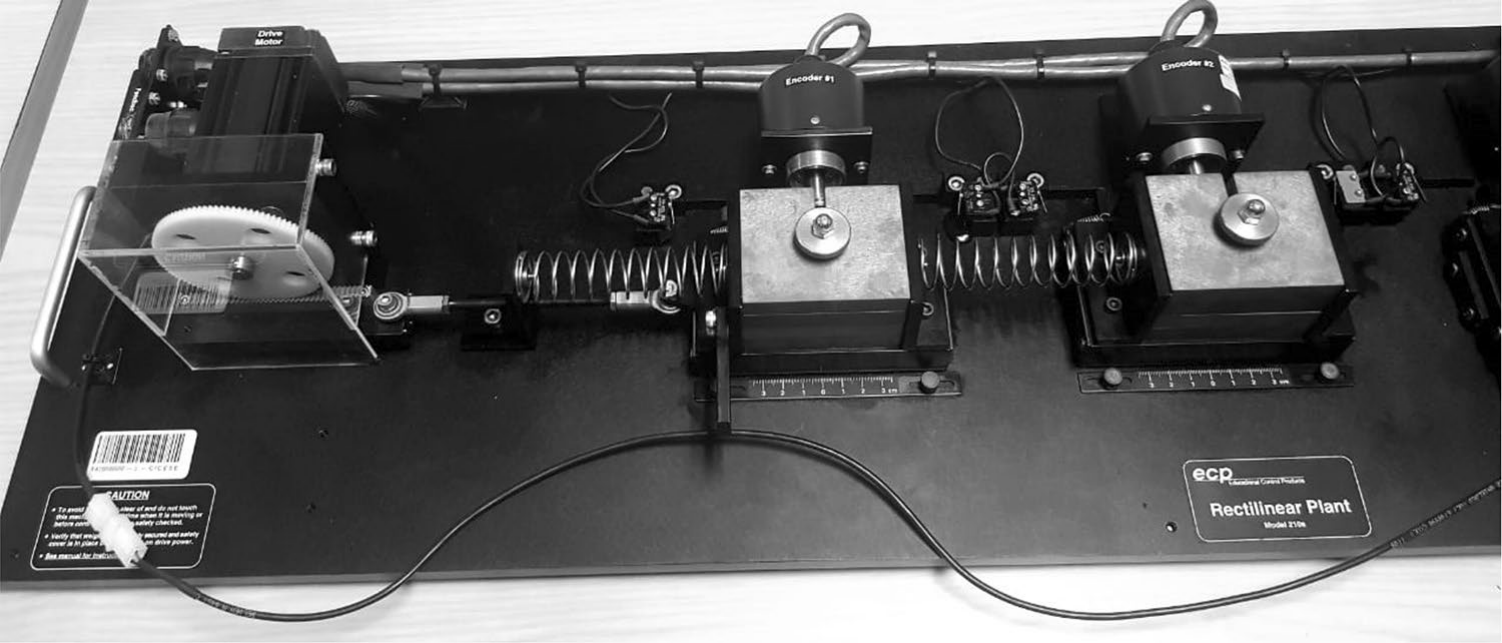
Rectilinear plant ECP model 210.

The experiments are conducted in the rectilinear plant ECP model 210, shown in Fig. 5, which consists of two mass-spring oscillators. The positions of the masses are obtained by means of encoders and one of the masses can be directly actuated by means of a servo actuator. The parameter values for the system and for the nonlinear term (5) are as given in Table 1 and a data acquisition card is used for measuring the signals from the encoders and also, for sending the nonlinear term (5) to the servo actuator. Furthermore, the velocity of mass one, which is required in (5), is obtained via a Luenberger observer.

**Figure 6:**
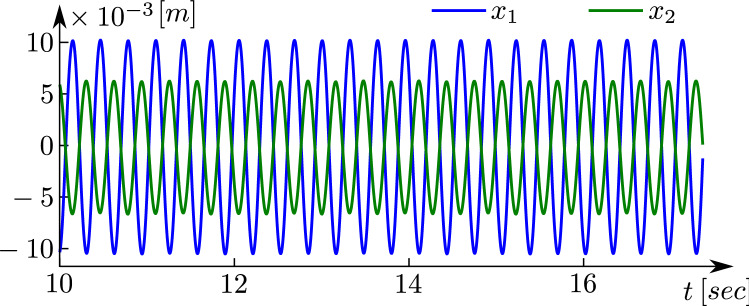
Experimental results. Time series for $x_{1}$ (blue) and $x_{2}$ (green).

The time series obtained from the experiment for $x_{1}$ (blue) and $x_{2}$ (green), are shown in Fig. 6. The amplitude of $x_{1}$ is $x_{1amp}=0.0105$ (m), whereas the amplitude for $x_{2}$ is $x_{2amp}=0.0065$ (m).

Note that the ratio of amplitudes $x_{1amp}/x_{2amp}=1.6180$ is very close to the golden number $\varphi =1.6180 \ldots$, there is just a tiny error of 0.8$\%$.

**Figure 7:**
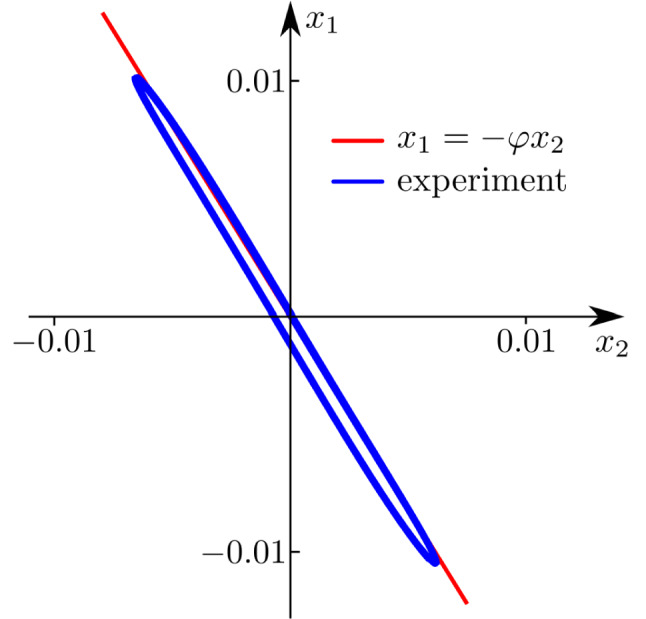
Projection of the time series $x_{1}(t),x_{2}(t)$ onto the ($x_{2},x_{1}$)-plane. Blue line: experiment. Red line: reference line with slope equal to $-\varphi$.

To further illustrate this, the measured time series are projected onto the ($x_{2},x_{1}$)-plane. This is shown in Fig. 7, where the blue line is the experimental result and the red line is an auxiliary line with slope equal to $-\varphi$. Note however, that the blue curve is not a perfect straight line due to the nonlinear effects like dry friction and the unavoidable differences between the oscillators. Nevertheless, the observed agreement is remarkable.

Now, for obtaining the frequency from the experimental data, it is convenient to take into account the following. Solutions $x_{i}$, $i=1,2$ are very well approximated by $x_i(t)=A_i\sin {{\tilde{\omega }} t}$, with ${\tilde{\omega }}$ the unknown frequency to be determined. Furthermore, the derivative of $x_{i}$ is ${\dot{x}}_{i}=B_{i}\cos {{\tilde{\omega }}t}$, with $B_{i}={\tilde{\omega }}A_{i}$. Thus, since the amplitude $A_{i}$ is already known, see Fig. 6, then the unknown frequency can be obtained by dividing the amplitude of the derivative between the amplitude of the signal, i.e. ${\tilde{\omega }}=B_{i}/A_{i}$.

In order to get $B_{i}$, the approximated derivative ${\mathscr {D}}x_i$ of $x_{i}$, also known as dirty derivative^26^, is computed as follows

$$\begin{aligned} {\mathscr {D}}x_i{:}{=}\frac{x_i(t_{n+1})-x_i(t_n)}{t_{n+1}-t_n}, \end{aligned}$$

for $n=1, \ldots ,m-1$, where *m* is the length of the signal. Note that this approximation holds for $t_{n+1}-t_n\ll 1$. Then, the value of $B_i$ is obtained from $B_i=\max ({\mathscr {D}}x_i)$. Using $x_{1}$ for computing the approximated derivative, it follows that $B_{1}=0.2166$. Consequently, the approximated oscillation frequency is ${\tilde{\omega }}=20.632$ (rad). On the other hand, the expected oscillation frequency, see Theorem 1, is $\varphi \omega =\varphi \sqrt{\frac{k}{m}}=20.936$ (rad).

Then there is an error of $1.45\%$ between the experimental and theoretical results, which is acceptable given the fact that the theoretical analysis has been conducted under the assumption of identical oscillators, which in practice is impossible to achieve.

**Figure 8:**
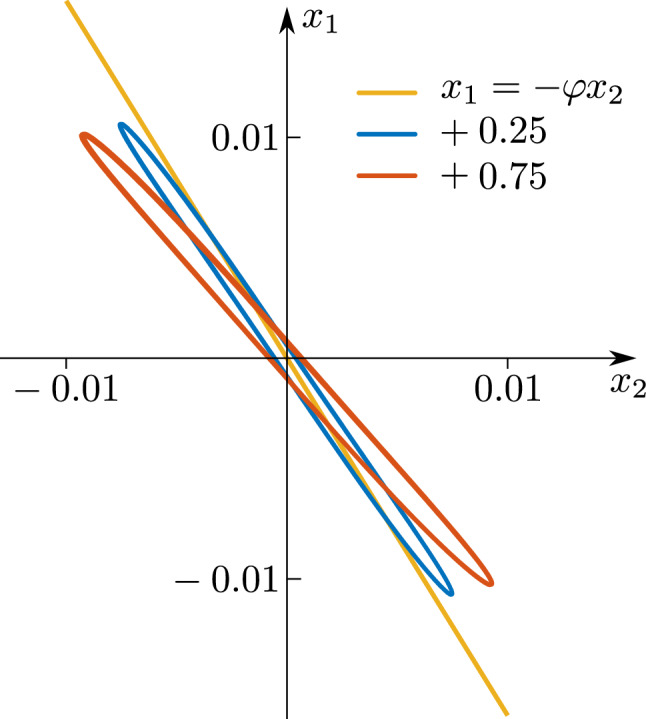
Experimental results for oscillators with different masses. Blue: mass difference of 0.250 (kg). Orange: mass difference of 0.750 (kg). The ratio of amplitudes deviates from the golden number (yellow line) as long as the mass differences increase. Thus the golden number can be used as a measure of how identical or dissimilar the oscillators are.

Additionally, in order to illustrate Remark 1, we have repeated the above experiment but this time the oscillators have different masses. Specifically, an extra mass is added to oscillator 1. The obtained results are shown in Fig. 8. The blue curve corresponds to the case that an extra mass of 0.250 (kg) is added, whereas the orange curve corresponds to an extra mass of 0.750 (kg). From this figure it is clear to see that as the difference in mass increases, the ratio of amplitudes deviates more and more from the golden number (yellow line).

Ultimately, the experimental results presented here give strong support to the theoretical and numerical results presented in previous sections.

## Discussion and conclusions

We have provided analytical, numerical, and experimental evidence that the golden number appears in the amplitude and frequency of the asymptotically stable periodic solution corresponding to an underactuated mechanical system with weakly nonlinear self-excitation.

For the case of *identical* oscillators, the absolute ratio of the amplitudes coincides exactly with the golden number and likewise, the oscillation frequency is given by the natural frequency of the oscillators scaled by the golden number. Interestingly, this is true independently of the value of the intrinsic parameters of the system like mass and stiffness, as long as the assumption of weak nonlinearity ($\epsilon \ll 1$) is satisfied.

Furthermore, it has been shown that if the ratio of amplitudes of the solutions of the coupled oscillators coincide with the golden number, then the system is *lossless*, i.e. there is no energy dissipation in the system.

On the other hand, the experimental results presented here have shown a remarkable approximation to the theoretical results besides the fact that the latter have been obtained assuming identical oscillators, whereas in the former this assumption is obviously never satisfied. In fact, while performing the experiments, we have noticed that for the system under consideration, the golden number can be used as a measure of how identical or dissimilar the oscillators are. Specifically, if the absolute value of the ratio of amplitudes is very close to the golden number then it is possible to conclude that the oscillators are practically identical without doing any extra computation.

Ultimately, based on the results reported in this manuscript, we claim that the mechanical system discussed here can be added to the list of systems/examples where the golden number emerges. Furthermore, since there exists a well-known relationship between mechanical and electrical circuits, see e.g. Ref.^27^, we believe that the results presented here may also be applicable to certain electronics circuits, like for example capacitively coupled LC circuits.

## Data Availability

The datasets generated during and/or analysed during the current study are available from the corresponding author on reasonable request.
